# Spinopelvic In Situ Fixation and Early Mobilization: A Case Report and Literature Review

**DOI:** 10.7759/cureus.36454

**Published:** 2023-03-21

**Authors:** Ali Alshehri, Hosam Alrehaili, Sultan Batayyib, Abdullah Saeed, Mohammed S Alharthi, Reem Alasmari

**Affiliations:** 1 Department of Orthopedic Surgery, King Abdulaziz Medical City, Riyadh, SAU; 2 Department of Orthopedic Surgery, National Guard Hospital, Riyadh, SAU; 3 Medicine and Surgery, College of Medicine, Imam Mohammad Ibn Saud Islamic University, Riyadh, SAU; 4 Medicine and Surgery, College of Medicine, Princess Nourah Bint Abdulrahman University, Riyadh, SAU

**Keywords:** sacroiliac screw, unstable pelvis, triangular osteosynthesis, spinopelvic fixation, spinopelvic dissociation

## Abstract

Pelvic fractures with sacroiliac extension are significant and complicated orthopedic injuries that pose a challenge in management and favorable outcomes. A 50-year-old obese female presented after a motor vehicle accident with pelvic fracture lateral compression. The patient underwent anterior external fixation with a left sacroiliac screw (SIS) on the next day of admission and was kept in a non-weight-bearing state. During her hospital stay, she developed deep vein thrombosis (DVT) and was treated. During the follow-up on the sixth week, the patient was not complying with her immobilization instructions and was exposing the left lower limb to weight bearing. The radiologic evaluation demonstrated a pulled-out SIS with a stable fracture. Considering that the patient was obese, had a history of DVT and COVID-19 infection, and the fracture was minimally displaced, it was decided to perform a spinopelvic in-situ fixation from L4 to S2 and augment it with a left SIS. The patient tolerated the surgery well and was referred to physiotherapy for early mobilization with full weight bearing. During her six-month and two-year follow-ups, she was well mobilized with no active complaints, and radiographic studies showed good healing, no displacement, no signs of instability, and a stable construct. Our case report presents a very rare and difficult but successful management of a fracture displacement in a non-compliant patient with one pulled-out screw through fast-tracked in situ spinopelvic fixation with early mobilization and full weight bearing. To our knowledge, this is one of the rare reports detailing a patient undergoing in situ spinopelvic fixation due to minimally displaced fracture with comorbidities such as obesity and DVT. Our report demonstrates the viability of accepting pulled-out screws, with respect to the patient’s health, the fracture’s geometry, a quick follow-up in situ spinopelvic fixation, early mobilization, full weight-bearing outcomes, and a lower risk for complications.

## Introduction

Pelvic ring injuries are relatively rare and uncommon injuries, occurring in 3-5% of cases, that most often result from significant trauma and can be associated with dislocation of the sacrum and sacroiliac joint which may have a tremendous impact on a patient’s quality of life [1]. In the past, most pelvic fractures were managed by skeletal traction and bed rest due to underdeveloped techniques and poor fixation devices, which meant that surgical interventions could yield unpredictable outcomes. However, new advances facilitated most orthopedic surgeons to become comfortable with the surgical treatment of fractures, with adequate fixation and early mobilization becoming more and more common [2].

Our case report presents a very rare and difficult but successful management of a fracture displacement in a non-compliant patient with pulled-out screws through fast-tracked in situ spinopelvic fixation with early mobilization and full weight bearing.

## Case presentation

**Figure 1:**
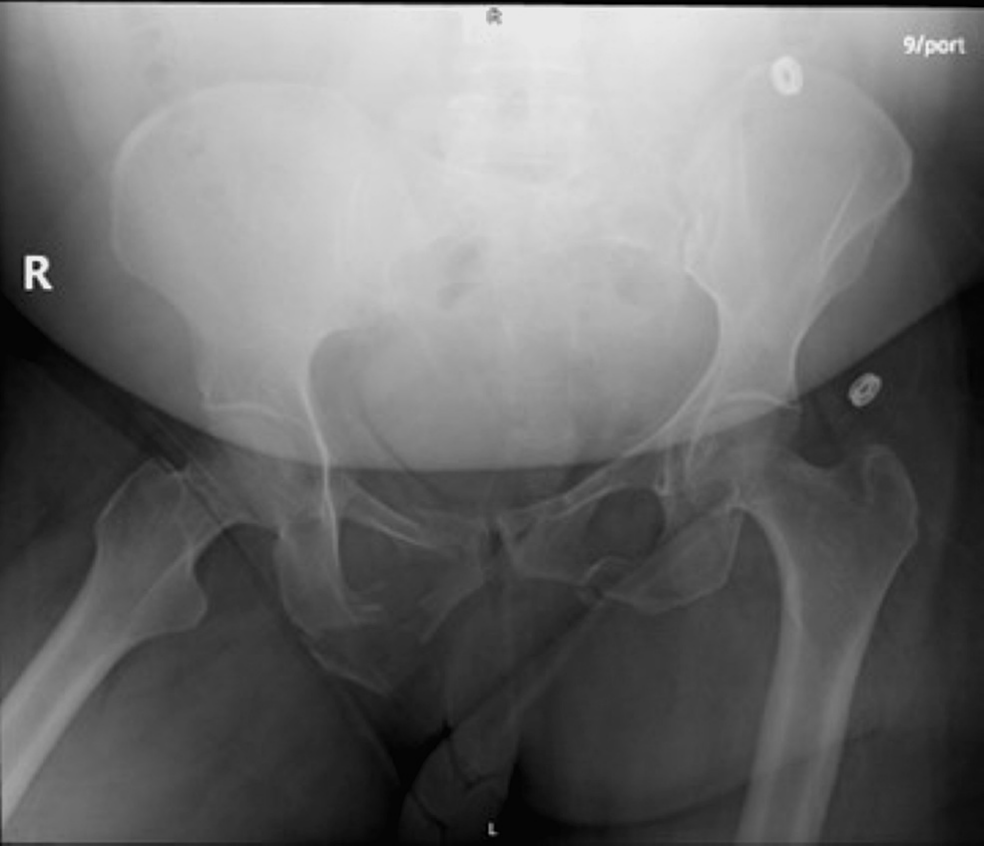
Radiograph showing resultant post-motor vehicle accident right superior and inferior pubic rami fracture with no extension to the sacrum, as well as left superior and inferior pubic rami with extension to the sacrum.

A 50-year-old woman presented to the emergency department following a motor vehicle accident (MVA), with a right superior and inferior pubic rami fracture with no extension to the sacrum and a left superior and inferior pubic rami with extension to sacrum through the neural foramina and sacroiliac joint (Figure 1) left lateral compression fracture.

Physical examination revealed that the patient was alert and oriented with a Glasgow Coma Scale score of 15/15. The pain scored 8/10 on the numeric pain rating scale. No neurological deficits were found with intact distal lower limb pulses, and the left hip range of motion was intact active and passive, with a mild decrease in muscle strength, especially hip flexors due to pain 4+/5 in power using the MRC grading. Additionally, the patient was found to have friction burn in the left upper flank area with a body mass index of 44 kg/m^2^.

**Figure 2:**
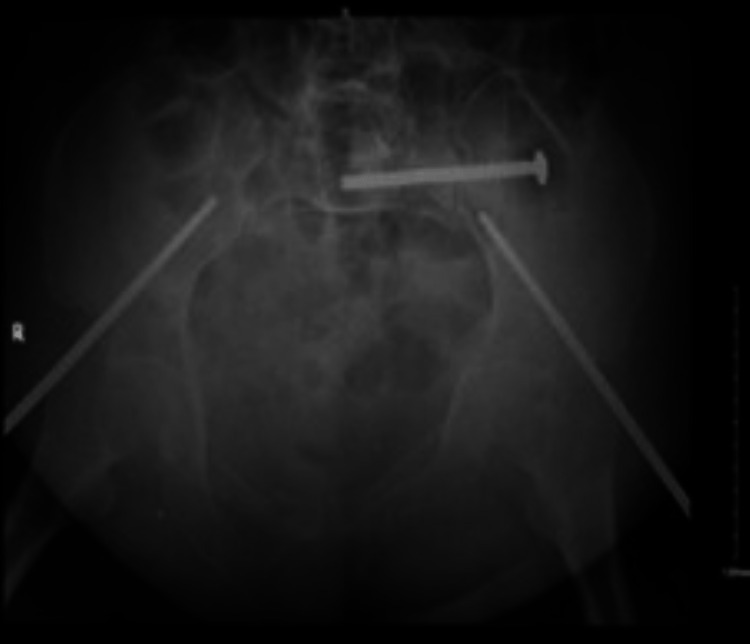
Radiograph demonstrating immediate postoperative anterior external fixator with a left sacroiliac screw.

The patient underwent an anterior external fixator with a left sacroiliac screw (SIS) on the next day of admission (Figure 2) and was kept in a non-weight-bearing state, bed-to-chair mobility, and instructed for daily pin-site dressing check.

After one week, the patient presented to the clinic. She was doing well with good compliance and immobilization with the non-weight bearing of left lower limbs, and wounds that appeared to be healing well. Pelvis X-rays obtained at that time revealed no signs of instability or implant complications.

**Figure 3:**
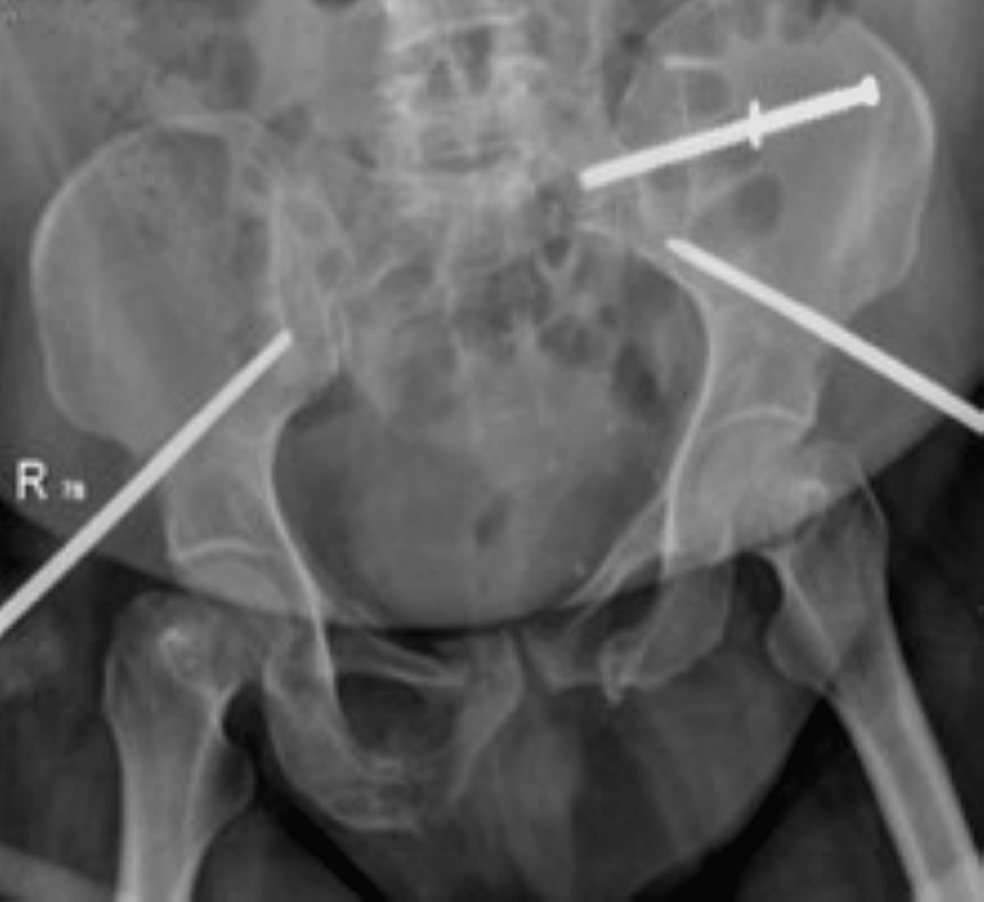
Radiograph demonstrating a pulled-out sacroiliac screw with a stable fracture on follow-up.

**Figure 4:**
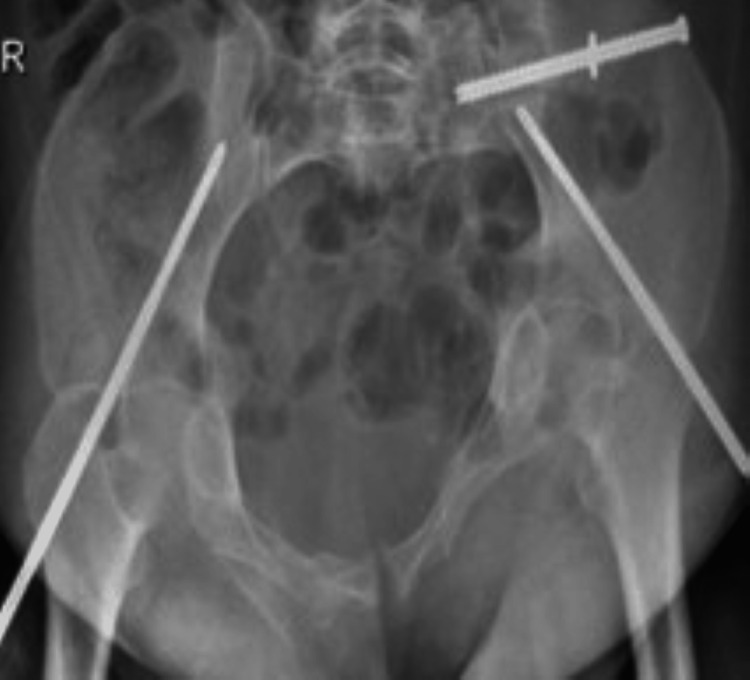
Radiograph demonstrating a pulled-out sacroiliac screw with a stable fracture on follow-up.

**Figure 5:**
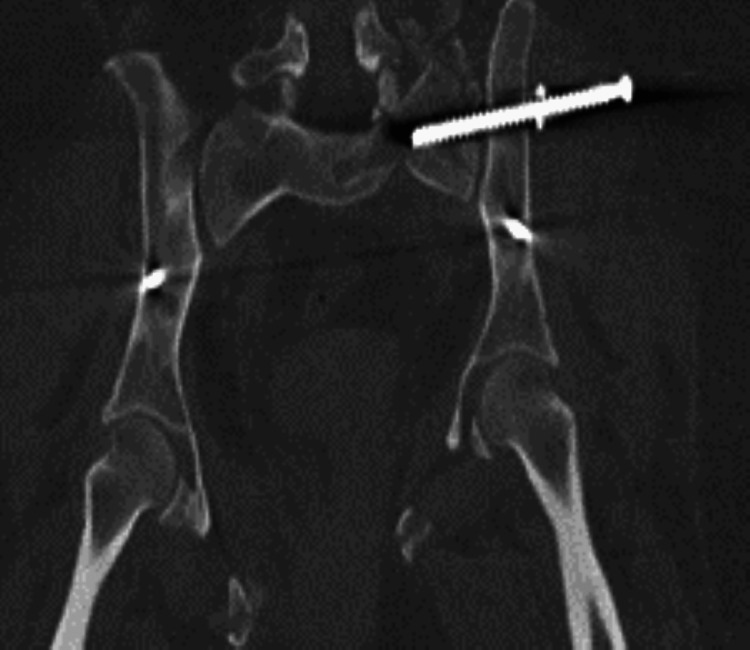
Radiograph demonstrating a pulled-out sacroiliac screw with a stable fracture on follow-up.

During the follow-up in the sixth week, the patient started to be non-compliant with her immobilization instructions, and she was bearing weight on the left lower limb. Follow-up X-rays showed pulled-out SIS with minimal displacement (Figures 3-5).

The patient was admitted and later developed COVID-19. SIS and anterior external fixator were removed, and left lower limb skeletal traction was applied. After discussing with the patient the surgical options for her case and the benefits and risks of spinopelvic in situ fixation with left SIS, the patient gave her consent to undergo surgery. Afterward, the fracture was stable clinically and radiologically with hard callus formation, and the patient tolerated the surgery well.

On postoperative day one, the patient was able to mobilize well and fully weight bear without any aids under physiotherapy guidance. In addition, postoperatively, she stayed as an inpatient for three days for pain control and physiotherapy and was discharged in good condition.

**Figure 6:**
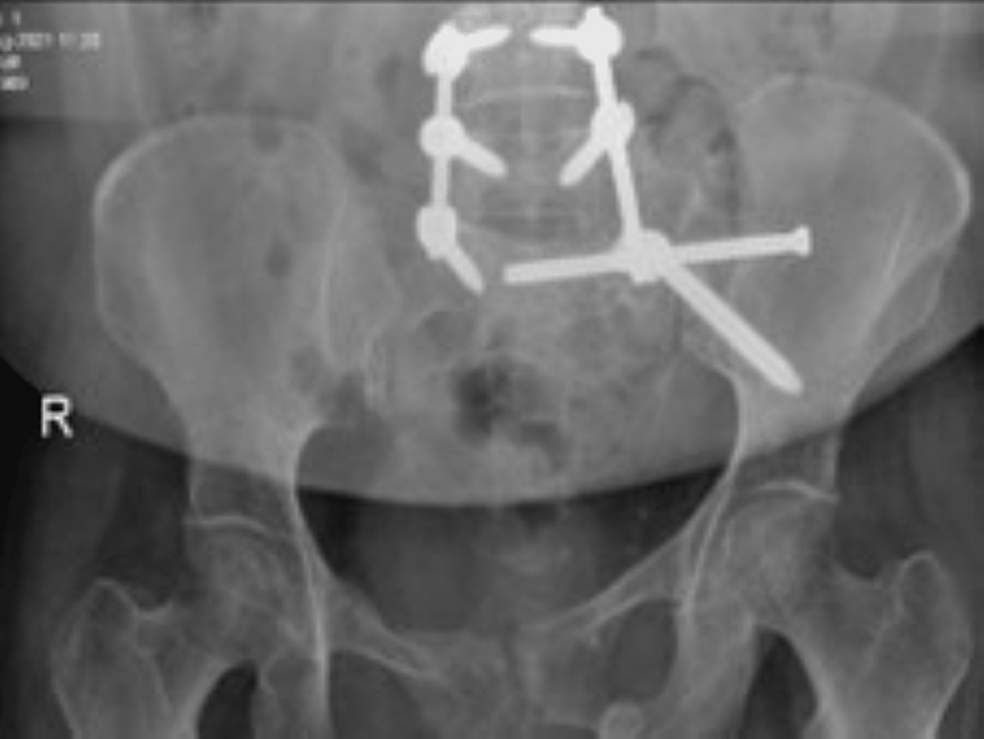
Radiograph demonstrating postoperative in situ spinopelvic fixation percutaneous sacroiliac screw fully threaded at S1 inserted.

**Figure 7:**
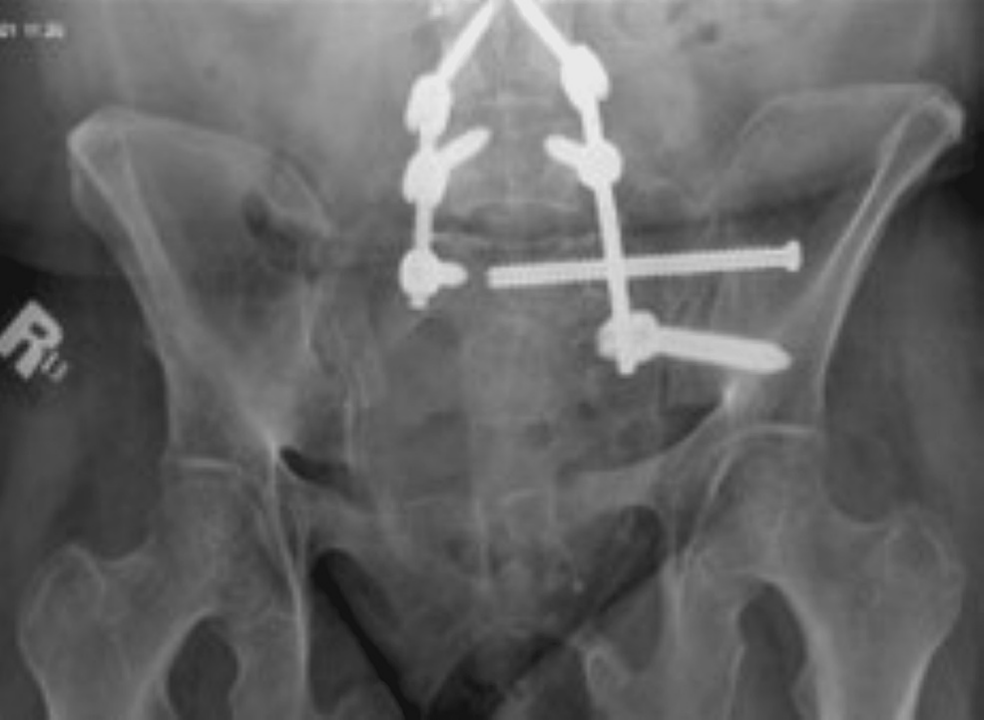
Radiograph demonstrating postoperative in situ spinopelvic fixation percutaneous sacroiliac screw fully threaded at S1 inserted.

**Figure 8:**
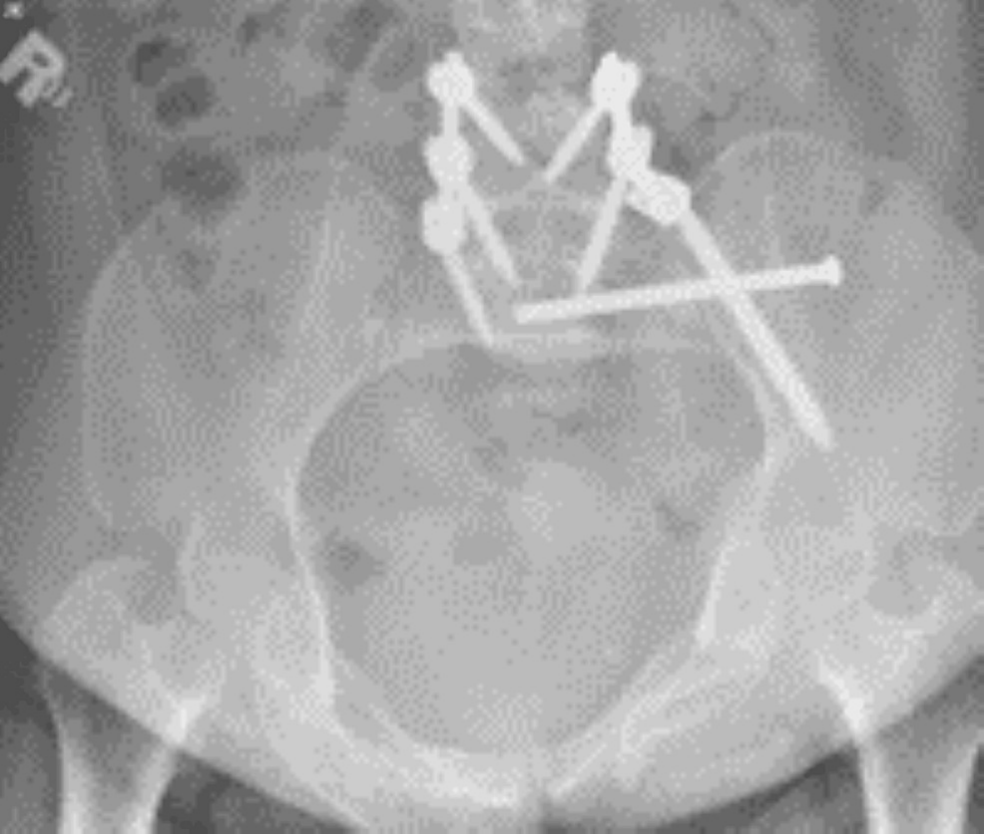
Radiograph demonstrating postoperative in situ spinopelvic fixation percutaneous sacroiliac screw fully threaded at S1 inserted.

After two weeks, the patient presented to the clinic for a wound check and was mobilizing well with no active complaints, and X-rays showed a stable fracture with no implant complications. She was fully mobilized following the last surgery (Figures 6-8).

Surgical technique

The patient was shifted to the operating room and was intubated in a prone position. Intraoperative X-rays showed a fracture in the same alignment with hard callus formation. The lower back left flank and hip were prepped and draped. The spine surgery team started the midline incision posteriorly with careful dissection to expose the posterior lumbar spine from L4 to the sacrum. Pedicle screws were inserted in L4, L5, right S1, and left S2. Subsequently, percutaneous SIS was inserted. A guidewire was inserted from the left flank to insert a cannulated screw 7.3 under fluoroscopy starting with a lateral view, Once the pelvis inlet and outlet views were in an acceptable position, the fully threaded S1 screw was successfully inserted. The spine team continued to insert rods and the wounds were closed with no complications and the patient remained stable.

Follow-up

The patient completed her surgical course in a good condition. She underwent physiotherapy at King Abdulaziz Medical City in Riyadh. Postoperatively, an assessment was done and distal neurovascular structures were intact. She was able to mobilize well rather than non-weight bearing of the left lower limb which was instructed immediately after the first surgery. Upon follow-up, the patient had no complaints with acceptable healing of her surgical wounds and a full range of motion. Moreover, she did not complain of any pain during her six-month follow-up, and the X-rays revealed a stable construct. After two years of surgery, along with physiotherapy, follow-up of the pelvis, and the lumbosacral spine X-rays revealed a satisfactory bone fusion without evidence of instability. The patient was mobilized well without walking aids and is currently patient on annual follow-up.

## Discussion

Spinopelvic fixation with SIS is a well-known method of fixation for an unstable pelvic fracture, with its own benefits and disadvantages that must be considered according to each case [3]. Fixation and repair of such injuries remain complex and challenging considering the difficult anatomy of the area and its unique biomechanical forces. Injuries in this area are catastrophic due to the significant effect on the patient’s quality of life [4].

Spinopelvic fixation using spinal instrumentation is a method of rigid fixation for unstable pelvic ring fractures [5]. A non-operative approach was the preferred choice of treatment for this type of fracture due to the lack of reliable surgical techniques and appropriate implants. However, this has changed over time with the advent of new techniques and the availability of new implants. Restoring the spinopelvic stability is the primary objective of surgical management of such fractures. Recent findings demonstrate that surgical treatment provides superior short and long-term benefits in comparison to conservative non-operative treatment [6-8]. High clinical suspicion, early lateral sacral radiographs, and pelvic computed tomography scans help in preventing delayed diagnosis. In addition to preventing progressive deformity and associated nerve root injury, surgical stabilization may help in the patient’s early mobilization from a reclining position [7].

## Conclusions

Spinopelvic dissociation is rare, with a high mechanism of injury. The management of such injuries is highly controversial and debated. In our case report, we realized that the fixation SIS and anterior external fixator may not be sufficient in obese and non-compliant patients. Early surgical stabilization is necessary to achieve good patient mobilization and obtain optimal clinical outcomes.
